# Supplementary material for: Climate change alters the future of natural floristic regions of deep evolutionary origins
Source: Nat Commun. 2024 Nov 2;15:9474. doi: 10.1038/s41467-024-53860-8 (PMC11531475; doi:10.1038/s41467-024-53860-8)
Supplement: Supplementary file 1 — Supplementary Information [file 41467_2024_53860_MOESM1_ESM.pdf]

Climate change alters the future of natural floristic regions of deep evolutionary origins

Samuel Minev-Benzecry and Barnabas H. Daru*

Corresponding author: bdaru@stanford.edu

This file includes:

Supplementary Figs. 1 to 9

Supplementary Tables 1 to 4

Supplementary Note 1

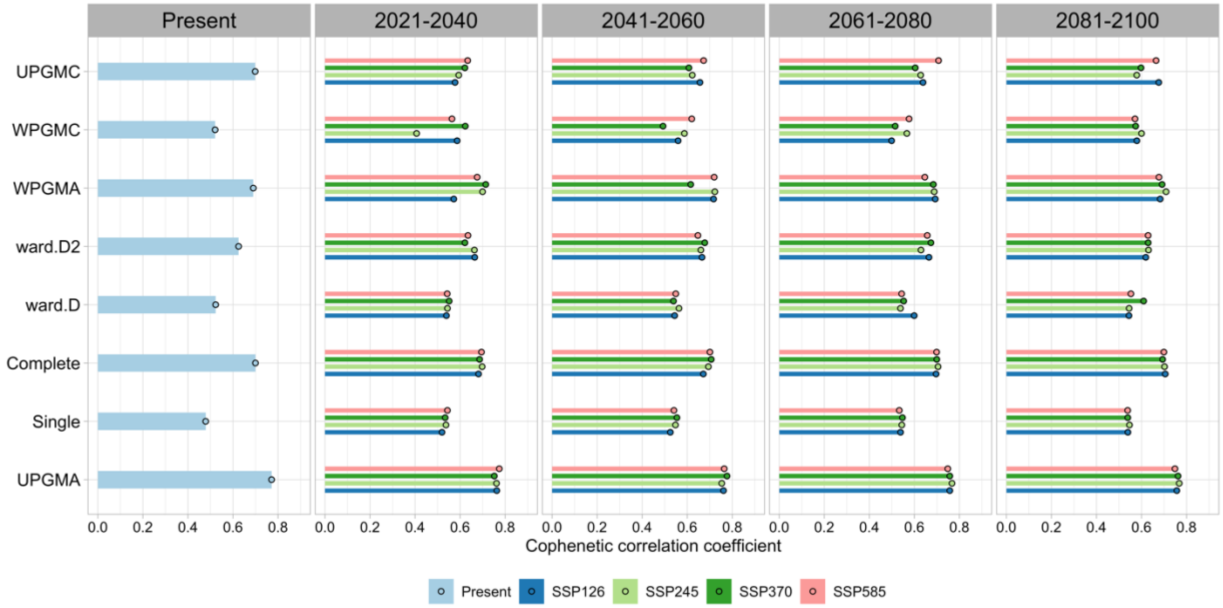

Supplementary Figure 1. Cluster algorithm selection and validation for clustering phylogenetic beta diversity of vascular plants under present and future climate scenarios. The performance of the clustering algorithms (n = 8) was quantified using cophenetic correlation coefficient which measures how well each algorithm fits the data, and ranges from 0 to 1. Individual data points are shown using overlaid dot plots. The UPGMA emerges as the best performing algorithm and was used to cluster matrices of Simpson's phylogenetic beta diversity. SSP shared socioeconomic pathways.

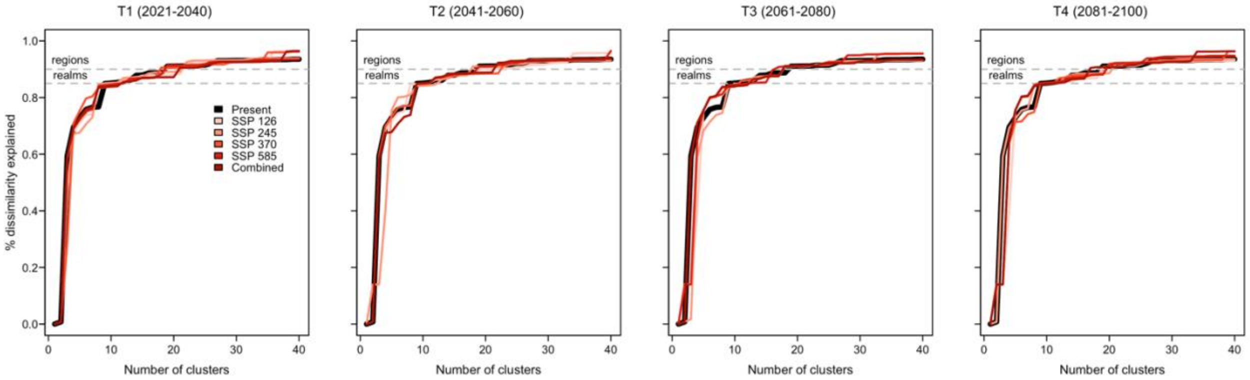

Supplementary Figure 2. Thresholds identifying the optimal number of clusters for delineating present and future floristic regions. Comparison of the cutoff points for identifying the optimal number of clusters for delineating present-day floristic regions (in black) versus future floristic regions under varying climate projections (in red). The matrices of Simpson's phylogenetic beta diversity ($p\beta_{sim}$) were agglomerated using unweighted pair group method with arithmetic averages (UPGMA). The cutoff points for the optimal number of clusters were identified based on their ability to explain 85% (all regions) and 90% (for realms) of the total sum of all the pβsim values that returned less than 40 clusters (i.e., species assemblages). The dashed horizontal lines indicate cutoff points where 85% and 90% of total $p\beta_{sim}$ is found between regions rather than within floristic regions.

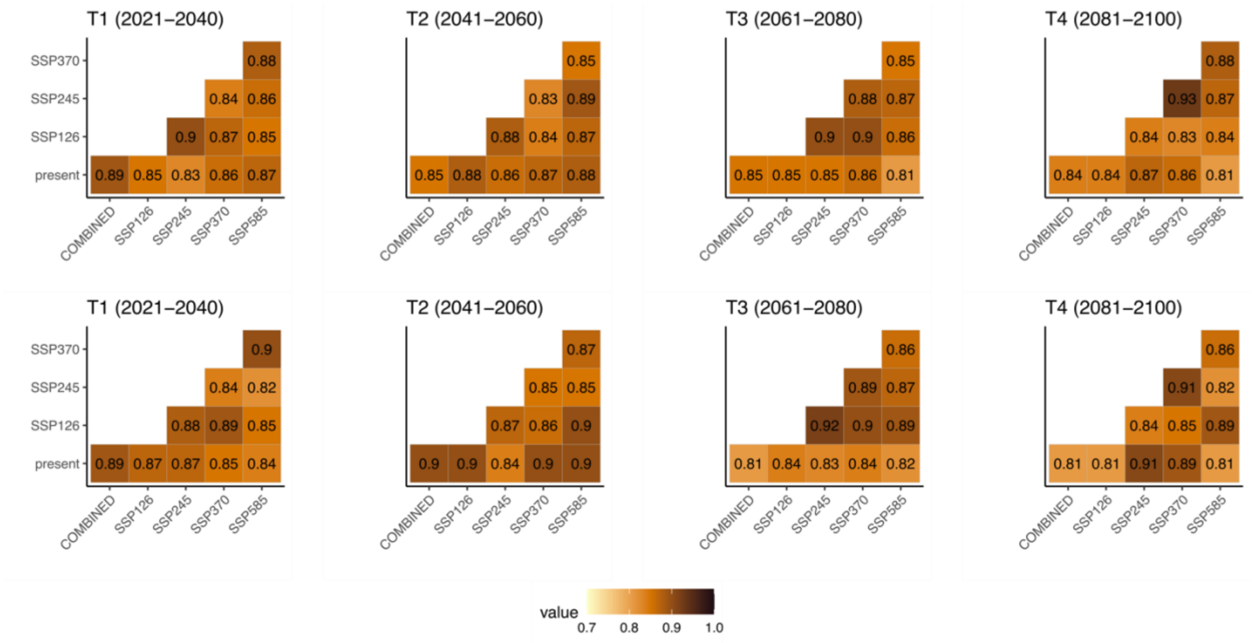

Supplementary Figure 3. V-measure statistics for spatial congruence of present-day biogeographic regions (first row) and realms (second row) with those projected under future climate scenarios for Simpson's phylogenetic beta diversity. The v-measure computes the degree of spatial association between regionalizations with scores ranging between 0 and 1 such that larger values indicate spatial congruence.

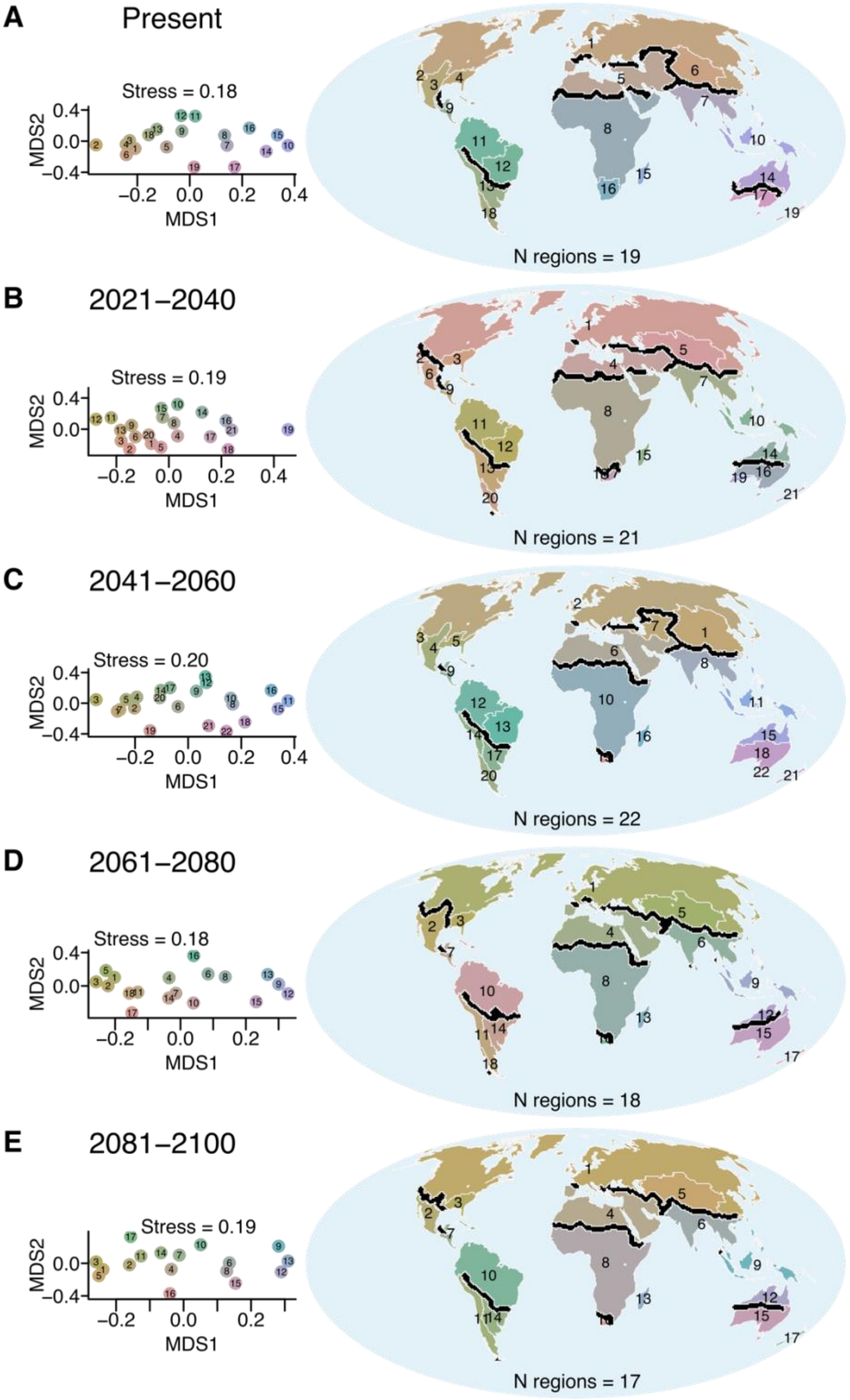

Supplementary Figure 4. Changes in vascular plant biogeographic regions under current and future climate scenarios in geographic space and in NMDS ordination space. (A) Global floristic regions of plants in the present-day delineated by clustering modelled range

maps for 189,269 vascular plant species with phylogenetic information and applying pairwise Simpson's β-diversity between 100 km × 100 km grid cells. Future floristic regions. Future species distributions were predicted by first modeling current plant distributions as a function of current environmental variables and then using this model to predict future plant distributions at new values of climate under different future scenarios and then using that to generate floristic regions for (B) T1: 2021-2040, (C) T2: 2041-2060, (D) T3: 2061-2080, and (E) T4: 2081-2100. The colors in the map and NMDS plots are identical and indicate levels of differentiation of the flora in different floristic regions such that floristic regions with similar colors have similar clades and those with different colors differ in the plant clades they enclose. Black lines separate floristic realms, while white lines separate floristic regions. The numbers in the map and NMDS plot are arbitrary and meant to identify clusters for each time period and do not represent a one-to-one match across time periods. The maps are in the equal-area World Mollweide projection.

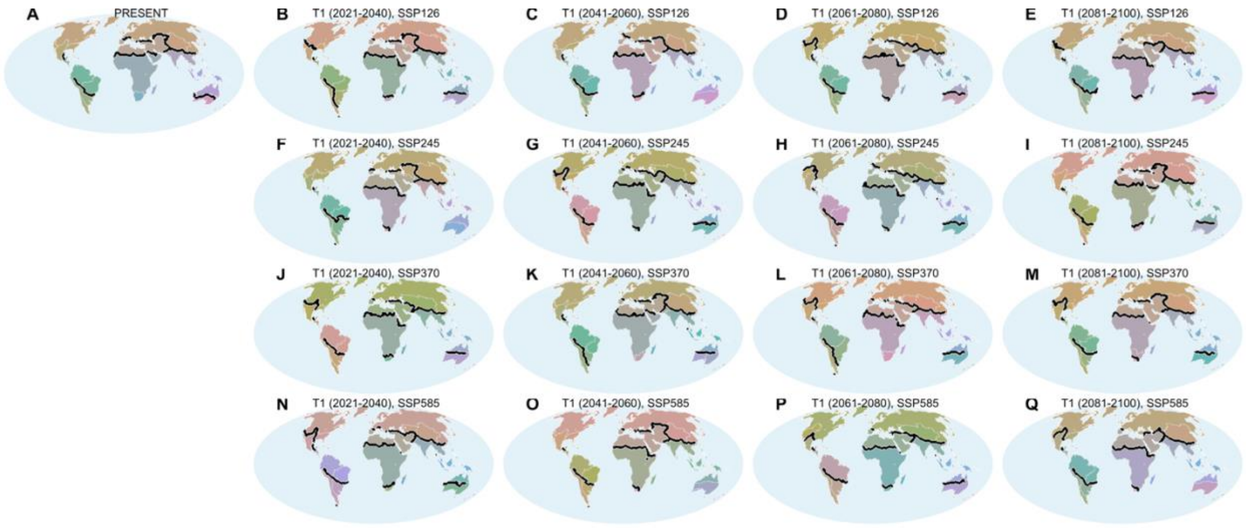

Supplementary Figure 5. Global floristic regions under present and future climate scenarios based on Simpson's phylogenetic beta diversity. (A) Global floristic regions of plants in the present-day delineated by clustering predicted range maps for 189,269 vascular plant species with phylogenetic information and applying pairwise phylogenetic dissimilarity between 100 km × 100 km grid cells. The colors indicate levels of differentiation of the flora in different floristic regions such that floristic regions with similar colors have similar clades and those with different colors differ in the plant clades they enclose. Black lines separate floristic realms, while white lines separate floristic regions. Future floristic regions projected under scenarios of climate change for (B) T1 (2021-2040) and SSP126, (C) T1 (2041-2060) and SSP126, (D) T1 (2061-2080) and SSP126, (E) T1 (2081-2100) and SSP126, (F) T1 (2021-2040) and SSP245, (G) T1 (2041-2060) and SSP245, (H) T1 (2061-2080) and SSP245, (I) T1 (2081-2100) and SSP245, (J) T1 (2021-2040) and SSP370, (K) T1 (2041-2060) and SSP370, (L) T1 (2061-2080) and SSP370, (M) T1 (2081-2100) and SSP370, (N) T1 (2021-2040) and SSP585, (O) T1 (2041-2060) and SSP585, (P) T1 (2061-2080) and SSP585, and (Q) T1 (2081-2100) and SSP585. The maps are in the equal-area World Mollweide projection.

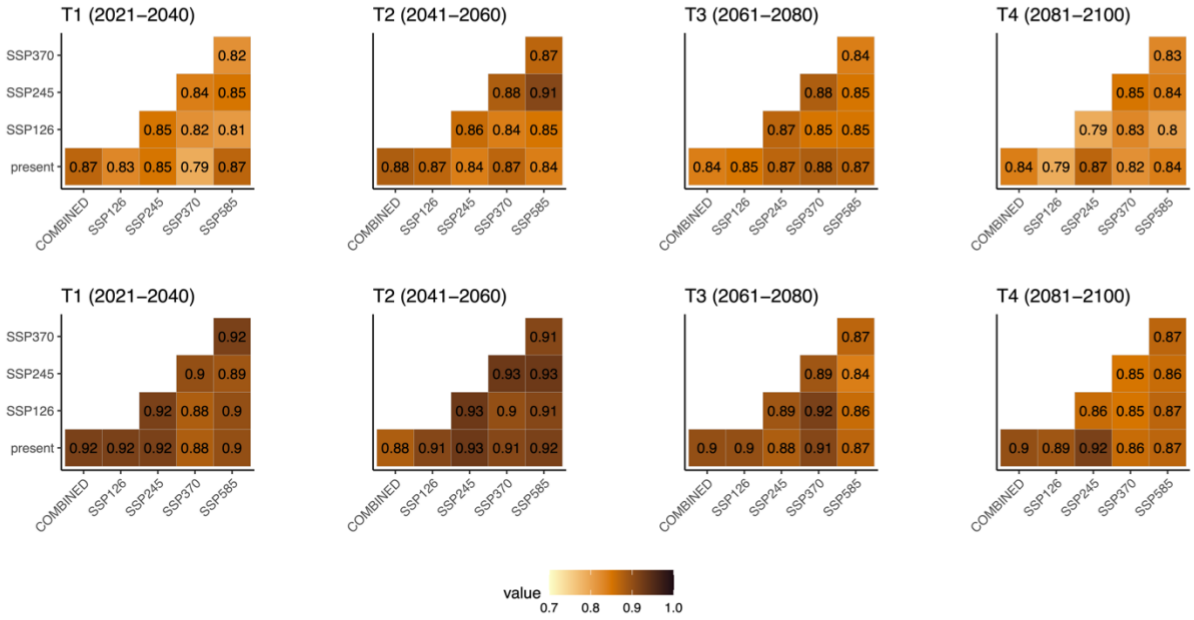

Supplementary Figure 6. V-measure statistics for spatial congruence of present-day biogeographic regions (first row) and realms (second row) with those projected under future climate scenarios for Sorensen's phylogenetic beta diversity. The v-measure computes the degree of spatial association between regionalizations with scores ranging between 0 and 1 such that larger values indicate spatial congruence.

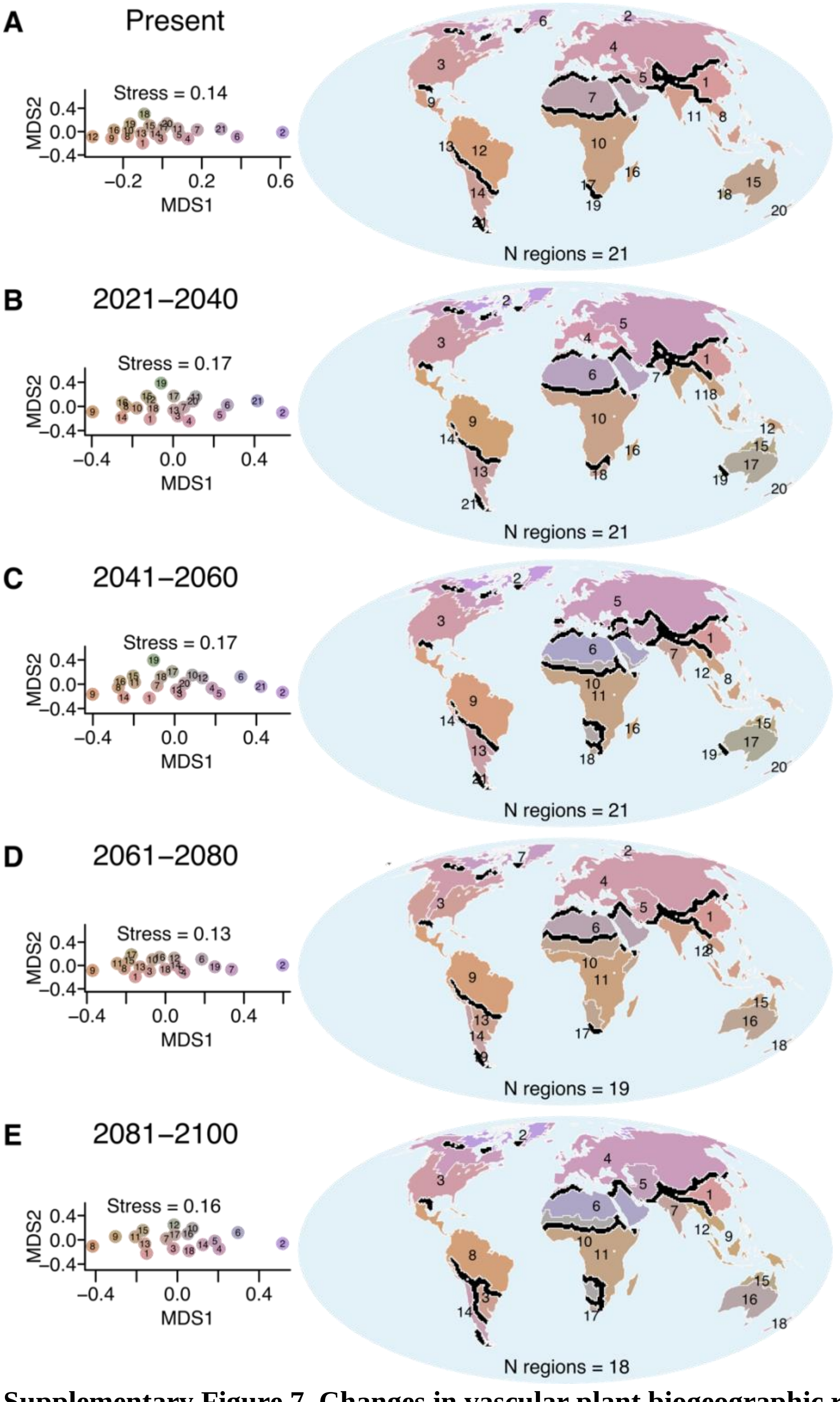

Supplementary Figure 7. Changes in vascular plant biogeographic regions under current and future climate scenarios in geographic space and in NMDS ordination space. (A) Global floristic regions of plants in the present-day delineated by clustering modelled range maps for 189,269 vascular plant species with phylogenetic information and applying pairwise

Sorensen's β-diversity between 100 km × 100 km grid cells. Future floristic regions: Future species distributions were predicted by first modeling current plant distributions as a function of current environmental variables and then using this model to predict future plant distributions at new values of climate under different future scenarios and then using that to generate floristic regions for (B) T1: 2021-2040, (C) T2: 2041-2060, (D) T3: 2061-2080, and (E) T4: 2081-2100. The colors in the map and NMDS plots are identical and indicate levels of differentiation of the flora in different floristic regions such that floristic regions with similar colors have similar clades and those with different colors differ in the plant clades they enclose. Black lines separate floristic realms, while white lines separate floristic regions. The numbers in the map and NMDS plot are arbitrary and meant to identify clusters for each time period and do not represent a one-to-one match across time periods. The maps are in the equal-area World Mollweide projection.

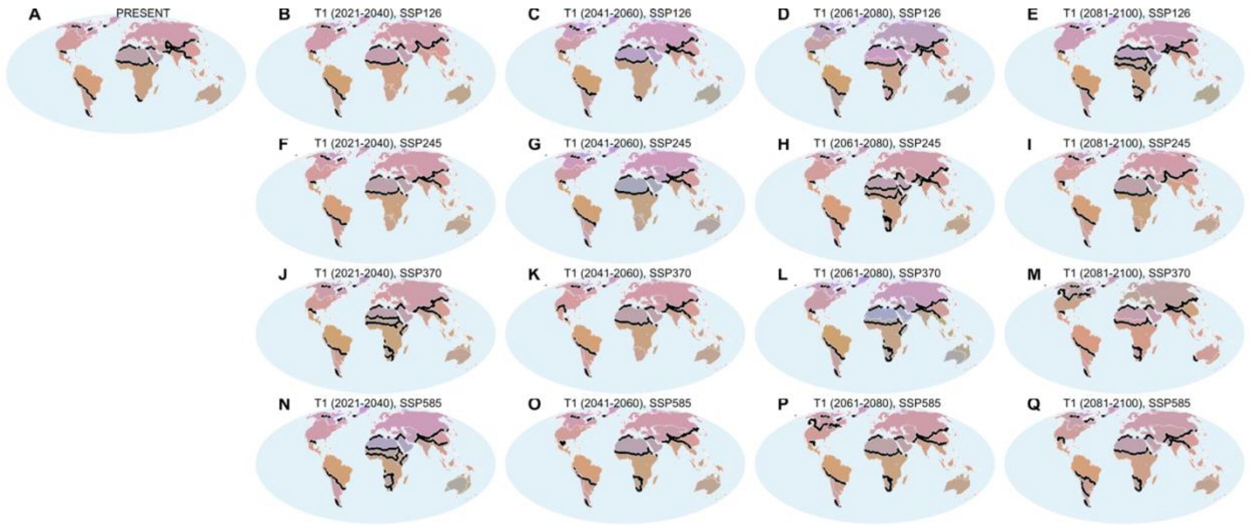

Supplementary Figure 8. Global floristic regions under present and future climate scenarios based on Sorensen's phylogenetic beta diversity. (A) Global floristic regions of plants in the present-day delineated by clustering predicted range maps for 189,269 vascular plant species with phylogenetic information and applying pairwise phylogenetic dissimilarity between 100 km × 100 km grid cells. The colors indicate levels of differentiation of the flora in different floristic regions such that floristic regions with similar colors have similar clades and those with different colors differ in the plant clades they enclose. Black lines separate floristic realms, while white lines separate floristic regions. Future floristic regions projected under scenarios of climate change for (B) T1 (2021-2040) and SSP126, (C) T1 (2041-2060) and SSP126, (D) T1 (2061-2080) and SSP126, (E) T1 (2081-2100) and SSP126, (F) T1 (2021-2040) and SSP245, (G) T1 (2041-2060) and SSP245, (H) T1 (2061-2080) and SSP245, (I) T1 (2081-2100) and SSP245, (J) T1 (2021-2040) and SSP370, (K) T1 (2041-2060) and SSP370, (L) T1 (2061-2080) and SSP370, (M) T1 (2081-2100) and SSP370, (N) T1 (2021-2040) and SSP585, (O) T1 (2041-2060) and SSP585, (P) T1 (2061-2080) and SSP585, and (Q) T1 (2081-2100) and SSP585. The maps are in the equal-area World Mollweide projection.

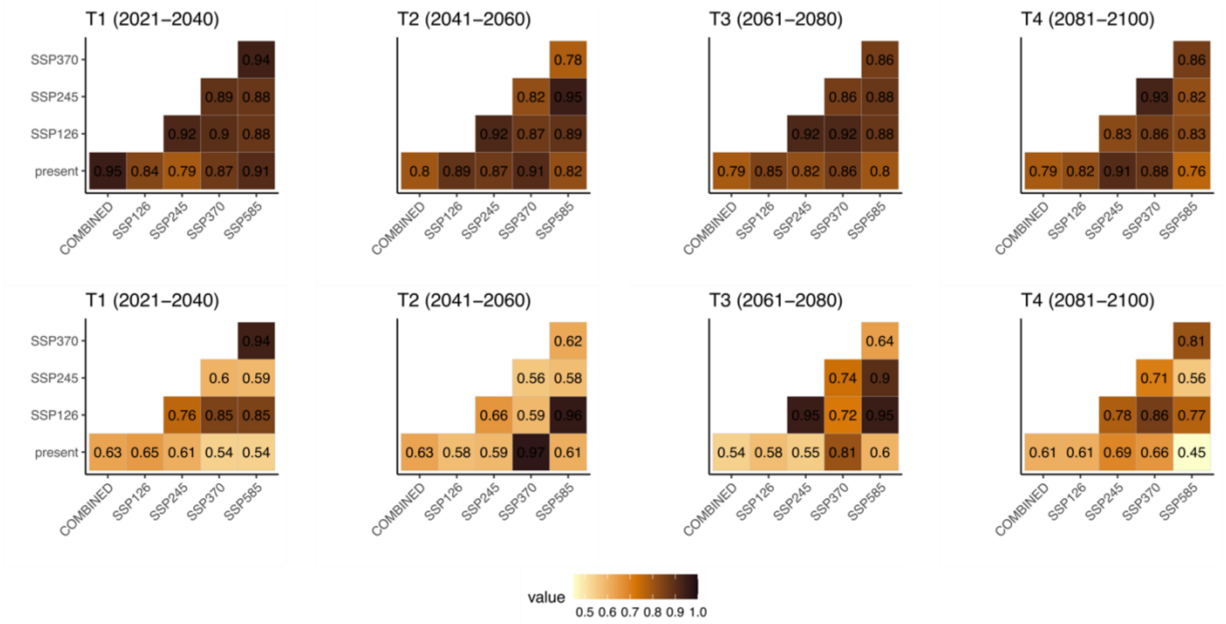

Supplementary Figure 9. Spatial correlation Simpson index. Spatial correlations among positions of terrestrial boundaries across the different regionalizations for floristic regions in the present day versus future climate scenarios clustered using Simpson's phylogenetic beta diversity. Top row indicate all region boundaries and bottom row corresponds to realm boundaries. The statistical test used is the Pearson's correlation corrected for spatial autocorrelation. Indicated are the correlation coefficients.

Supplementary Table 1. Changes in the structure of biogeographic regions under climate change. Shifts in Simpson's phylogenetic beta diversity assessed by comparing the grid-cell compositional dissimilarity for delineating present vs future floristic regions when considering β-diversity within present-day biogeographic regions. The analysis was conducted by using a two-sided *t*-test, followed by Cohen's *d*, with 1000 bootstrap replicates to estimate effect sizes. Data are presented as Cohen's *d* ranging from 0 (no effect) to +1 or -1 (large effect), with positive values indicating differentiation, whereas negative values indicate homogenization.

current	future	P value	t	cluster	Cohens *d*	N cur	N fut	CI lower	CI upper	year	region
0.44	0.44	0.799	-0.25	1	0.00	5280	5280	-0.05	0.03	2021-2040	Circumboreal 1
0.53	0.53	0.723	0.36	2	0.06	65	65	-0.3	0.43	2021-2040	Californian 2
0.49	0.49	0.557	0.59	3	0.05	309	309	-0.1	0.21	2021-2040	Madrean 3
0.50	0.50	0.156	-1.42	4	-0.15	170	170	-0.36	0.06	2021-2040	N American Atlantic 4
0.48	0.48	0.173	1.36	5	0.05	1521	1521	-0.02	0.12	2021-2040	Saharo Arabian 5
0.49	0.49	0.071	1.81	6	0.10	664	664	-0.005	0.21	2021-2040	Turanian-Tibetan 6
0.53	0.53	0.317	1.00	7	0.06	648	648	-0.06	0.17	2021-2040	Indian-Indochinese 7
0.52	0.52	0.975	0.03	8	0.00	2456	2456	-0.06	0.06	2021-2040	Afrotropic 8
0.53	0.53	0.715	-0.37	9	-0.04	161	161	-0.26	0.17	2021-2040	Caribbean 9
0.60	0.60	0.728	-0.35	10	-0.02	479	479	-0.15	0.11	2021-2040	Malesian 10
0.58	0.58	0.019	2.36	11	0.12	783	783	0.03	0.22	2021-2040	Amazonian 11
0.59	0.59	0.232	-1.20	12	-0.07	516	516	-0.21	0.04	2021-2040	Brazilian 12
0.54	0.54	0.922	0.10	13	0.01	392	392	-0.14	0.15	2021-2040	Andean-Chilean 13
0.60	0.60	0.329	-0.98	14	-0.06	484	484	-0.18	0.06	2021-2040	Monsoonal Tropics-Eremean 14
0.59	0.59	0.763	-0.30	15	-0.05	74	74	-0.38	0.28	2021-2040	Madagascan 15
0.57	0.57	0.589	0.54	16	0.06	189	189	-0.14	0.26	2021-2040	South African 16
0.61	0.61	0.947	-0.07	17	0.00	360	360	-0.15	0.15	2021-2040	South Australian 17
0.55	0.55	0.996	0.00	18	0.00	177	177	-0.2	0.22	2021-2040	Patagonian 18
0.61	0.62	0.350	-0.94	19	-0.19	47	47	-0.68	0.2	2021-2040	Neozylandic 19
0.44	0.45	0.098	-1.66	1	-0.03	5280	5280	-0.07	0.0051	2041-2060	Circumboreal 1
0.53	0.53	0.748	-0.32	2	-0.06	65	65	-0.44	0.32	2041-2060	Californian 2
0.49	0.49	0.872	-0.16	3	-0.01	309	309	-0.17	0.14	2041-2060	Madrean 3
0.50	0.50	0.107	-1.61	4	-0.18	170	170	-0.38	0.04	2041-2060	N American Atlantic 4
0.48	0.48	0.321	0.99	5	0.04	1521	1521	-0.03	0.11	2041-2060	Saharo Arabian 5
0.49	0.49	0.579	0.55	6	0.03	664	664	-0.08	0.13	2041-2060	Turanian-Tibetan 6
0.53	0.53	0.588	0.54	7	0.03	648	648	-0.08	0.14	2041-2060	Indian-Indochinese 7
0.52	0.52	0.696	-0.39	8	-0.01	2456	2456	-0.07	0.04	2041-2060	Afrotropic 8
0.53	0.53	0.545	-0.61	9	-0.07	161	161	-0.3	0.15	2041-2060	Caribbean 9
0.60	0.60	0.488	-0.69	10	-0.04	479	479	-0.17	0.1	2041-2060	Malesian 10
0.58	0.57	0.001	3.43	11	0.17	783	783	0.08	0.28	2041-2060	Amazonian 11
0.59	0.59	0.098	-1.65	12	-0.10	516	516	-0.23	0.02	2041-2060	Brazilian 12
0.54	0.55	0.444	-0.77	13	-0.05	392	392	-0.19	0.09	2041-2060	Andean-Chilean 13
0.60	0.60	0.134	-1.50	14	-0.10	484	484	-0.23	0.02	2041-2060	Monsoonal Tropics-Eremean 14
0.59	0.59	0.203	-1.28	15	-0.21	74	74	-0.55	0.09	2041-2060	Madagascan 15
0.57	0.57	0.957	-0.05	16	-0.01	189	189	-0.21	0.2	2041-2060	South African 16
0.61	0.61	0.786	-0.27	17	-0.02	360	360	-0.16	0.13	2041-2060	South Australian 17
0.55	0.55	0.766	-0.30	18	-0.03	177	177	-0.23	0.19	2041-2060	Patagonian 18
0.61	0.62	0.062	-1.89	19	-0.39	47	47	-0.95	0.03	2041-2060	Neozylandic 19
0.44	0.45	0.026	-2.22	1	-0.04	5280	5280	-0.08	-0.007	2061-2080	Circumboreal 1
0.53	0.53	0.513	0.66	2	0.12	65	65	-0.25	0.45	2061-2080	Californian 2
0.49	0.49	0.600	0.53	3	0.04	309	309	-0.12	0.2	2061-2080	Madrean 3
0.50	0.50	0.071	-1.81	4	-0.20	170	170	-0.39	0.0059	2061-2080	N American Atlantic 4
0.48	0.48	0.091	1.69	5	0.06	1521	1521	-0.01	0.13	2061-2080	Saharo Arabian 5
0.49	0.49	0.341	0.95	6	0.05	664	664	-0.06	0.16	2061-2080	Turanian-Tibetan 6

0.53	0.53	0.217	1.24	7	0.07	648	648	-0.04	0.18	2061-2080	Indian-Indochinese 7
0.52	0.52	0.743	-0.33	8	-0.01	2456	2456	-0.07	0.05	2061-2080	Afrotropic 8
0.53	0.53	0.981	0.02	9	0.00	161	161	-0.22	0.23	2061-2080	Caribbean 9
0.60	0.60	0.232	-1.20	10	-0.08	479	479	-0.21	0.07	2061-2080	Malesian 10
0.58	0.57	0.000	4.83	11	0.24	783	783	0.14	0.35	2061-2080	Amazonian 11
0.59	0.59	0.214	-1.24	12	-0.08	516	516	-0.2	0.05	2061-2080	Brazilian 12
0.54	0.54	0.764	-0.30	13	-0.02	392	392	-0.16	0.11	2061-2080	Andean-Chilean 13
0.60	0.61	0.027	-2.22	14	-0.14	484	484	-0.28	-0.01	2061-2080	Monsoonal Tropics-Eremean 14
0.59	0.59	0.057	-1.92	15	-0.32	74	74	-0.66	0.0085	2061-2080	Madagascan 15
0.57	0.57	0.709	0.37	16	0.04	189	189	-0.16	0.25	2061-2080	South African 16
0.61	0.61	0.920	-0.10	17	-0.01	360	360	-0.16	0.14	2061-2080	South Australian 17
0.55	0.55	0.998	0.00	18	0.00	177	177	-0.2	0.2	2061-2080	Patagonian 18
0.61	0.62	0.026	-2.27	19	-0.47	47	47	-0.98	-0.04	2061-2080	Neozylandic 19
0.44	0.45	0.000	-4.96	1	-0.10	5280	5280	-0.13	-0.06	2081-2100	Circumboreal 1
0.53	0.53	0.548	0.60	2	0.11	65	65	-0.26	0.43	2081-2100	Californian 2
0.49	0.49	0.845	0.20	3	0.02	309	309	-0.15	0.18	2081-2100	Madrean 3
0.50	0.50	0.026	-2.23	4	-0.24	170	170	-0.46	-0.02	2081-2100	N American Atlantic 4
0.48	0.48	0.075	1.78	5	0.06	1521	1521	-0.01	0.14	2081-2100	Saharo Arabian 5
0.49	0.49	0.763	-0.30	6	-0.02	664	664	-0.13	0.09	2081-2100	Turanian-Tibetan 6
0.53	0.53	0.300	1.04	7	0.06	648	648	-0.05	0.17	2081-2100	Indian-Indochinese 7
0.52	0.52	0.620	-0.50	8	-0.01	2456	2456	-0.07	0.04	2081-2100	Afrotropic 8
0.53	0.53	0.726	0.35	9	0.04	161	161	-0.19	0.27	2081-2100	Caribbean 9
0.60	0.60	0.376	-0.89	10	-0.06	479	479	-0.18	0.08	2081-2100	Malesian 10
0.58	0.57	0.000	5.62	11	0.28	783	783	0.18	0.39	2081-2100	Amazonian 11
0.59	0.59	0.220	-1.23	12	-0.08	516	516	-0.19	0.04	2081-2100	Brazilian 12
0.54	0.55	0.178	-1.35	13	-0.10	392	392	-0.24	0.05	2081-2100	Andean-Chilean 13
0.60	0.61	0.008	-2.64	14	-0.17	484	484	-0.29	-0.04	2081-2100	Monsoonal Tropics-Eremean 14
0.59	0.59	0.029	-2.20	15	-0.36	74	74	-0.72	-0.04	2081-2100	Madagascan 15
0.57	0.57	0.520	0.64	16	0.07	189	189	-0.13	0.27	2081-2100	South African 16
0.61	0.61	0.839	-0.20	17	-0.02	360	360	-0.17	0.13	2081-2100	South Australian 17
0.55	0.55	0.470	-0.72	18	-0.08	177	177	-0.29	0.13	2081-2100	Patagonian 18
0.61	0.62	0.013	-2.54	19	-0.52	47	47	-1.02	-0.09	2081-2100	Neozylandic 19

Supplementary Table 2. Spatial correlations among positions of terrestrial boundaries across the different regionalizations for floristic regions. The statistical test used is the Pearson's correlation corrected for spatial autocorrelation. Indicated are the correlation coefficients, p values, estimated degrees of freedom (e.d.f) and estimated effective sample size (E.S.S.). All p-values correspond to two-sided tests unless otherwise specified.

Time horizon	scenario1	scenario2	Correlation coefficient	P value	edf	ESS
2021-2040	present	SSP126	0.84	3.42E-19	67.47	69.47
	present	SSP245	0.79	3.22E-16	67.43	69.43
	present	SSP370	0.87	2.39E-21	65.27	67.27
	present	SSP585	0.91	3.71E-25	60.76	62.76
	present	COMBINED	0.95	2.05E-27	52.88	54.88
	SSP126	SSP245	0.92	4.63E-31	72.30	74.30
	SSP126	SSP370	0.90	9.89E-29	74.75	76.75
	SSP126	SSP585	0.88	1.62E-24	72.15	74.15
	SSP245	SSP370	0.89	6.23E-27	74.89	76.89
	SSP245	SSP585	0.88	4.64E-25	71.81	73.81
	SSP370	SSP585	0.94	3.23E-33	68.75	70.75
2041-2060	present	SSP126	0.89	2.15E-24	64.94	66.94
	present	SSP245	0.87	1.07E-19	59.40	61.40
	present	SSP370	0.91	2.87E-24	58.04	60.04
	present	SSP585	0.82	2.74E-19	72.72	74.72
	present	COMBINED	0.80	6.86E-16	64.01	66.01
	SSP126	SSP245	0.92	9.14E-29	67.02	69.02
	SSP126	SSP370	0.87	6.95E-22	66.53	68.53
	SSP126	SSP585	0.89	1.47E-28	80.37	82.37
	SSP245	SSP370	0.82	1.06E-16	62.25	64.25
	SSP245	SSP585	0.95	6.39E-37	70.21	72.21
	SSP370	SSP585	0.78	2.85E-17	75.12	77.12
2061-2080	present	SSP126	0.85	3.36E-18	59.49	61.49

	present	SSP245	0.82	2.93E-15	57.53	59.53
	present	SSP370	0.86	1.47E-23	74.60	76.60
	present	SSP585	0.80	6.04E-15	60.45	62.45
	present	COMBINED	0.79	1.70E-15	63.91	65.91
	SSP126	SSP245	0.92	1.02E-24	57.03	59.03
	SSP126	SSP370	0.92	3.83E-34	78.49	80.49
	SSP126	SSP585	0.88	7.54E-22	63.53	65.53
	SSP245	SSP370	0.86	2.39E-24	76.60	78.60
	SSP245	SSP585	0.88	2.33E-21	59.75	61.75
	SSP370	SSP585	0.86	2.49E-24	77.34	79.34
2081-2100	present	SSP126	0.82	3.41E-18	66.89	68.89
	present	SSP245	0.91	3.92E-24	59.48	61.48
	present	SSP370	0.88	8.28E-28	78.92	80.92
	present	SSP585	0.76	5.21E-14	66.47	68.47
	present	COMBINED	0.79	1.20E-15	66.21	68.21
	SSP126	SSP245	0.83	3.54E-19	67.71	69.71
	SSP126	SSP370	0.86	9.40E-29	90.87	92.87
	SSP126	SSP585	0.83	7.22E-23	82.35	84.35
	SSP245	SSP370	0.93	2.02E-36	79.22	81.22
	SSP245	SSP585	0.82	4.53E-18	67.75	69.75
	SSP370	SSP585	0.86	4.90E-27	85.76	87.76

Supplementary Table 3. Spatial correlations among positions of terrestrial boundaries across the different regionalizations for floristic realms. The statistical test used is the Pearson's correlation corrected for spatial autocorrelation. Indicated are the correlation coefficients, p values, estimated degrees of freedom (e.d.f) and estimated effective sample size (E.S.S.). All p-values correspond to two-sided tests unless otherwise specified.

Time horizon	scenario1	scenario2	Correlation coefficient	P value	edf	ESS
2021-2040	present	SSP126	0.65	9.67E-05	28.06	30.06
	present	SSP245	0.61	9.57E-04	24.33	26.33
	present	SSP370	0.54	5.29E-04	34.92	36.92
	present	SSP585	0.54	4.27E-04	36.58	38.58
	present	COMBINED	0.63	1.40E-04	29.40	31.40
	SSP126	SSP245	0.76	5.65E-09	39.80	41.80
	SSP126	SSP370	0.85	2.84E-14	44.75	46.75
	SSP126	SSP585	0.85	1.13E-13	42.92	44.92
	SSP245	SSP370	0.60	7.34E-07	55.23	57.23
	SSP245	SSP585	0.59	2.73E-07	61.20	63.20
	SSP370	SSP585	0.94	1.48E-22	43.88	45.88
2041-2060	present	SSP126	0.58	1.05E-03	26.63	28.63
	present	SSP245	0.59	1.70E-04	33.87	35.87
	present	SSP370	0.97	8.66E-10	13.36	15.36
	present	SSP585	0.61	7.53E-04	25.08	27.08
	present	COMBINED	0.63	5.03E-04	24.64	26.64
	SSP126	SSP245	0.66	2.40E-08	55.70	57.70
	SSP126	SSP370	0.59	9.97E-04	25.45	27.45
	SSP126	SSP585	0.96	1.70E-09	14.42	16.42
	SSP245	SSP370	0.56	4.35E-04	33.95	35.95
	SSP245	SSP585	0.58	4.52E-07	62.27	64.27
	SSP370	SSP585	0.62	7.24E-04	23.61	25.61
2061-2080	present	SSP126	0.58	1.71E-04	35.45	37.45

	present	SSP245	0.55	6.16E-04	32.91	34.91
	present	SSP370	0.81	1.99E-06	21.67	23.67
	present	SSP585	0.60	1.49E-04	32.26	34.26
	present	COMBINED	0.54	6.67E-04	33.84	35.84
	SSP126	SSP245	0.95	6.69E-24	43.63	45.63
	SSP126	SSP370	0.72	2.20E-07	37.39	39.39
	SSP126	SSP585	0.95	5.25E-26	47.14	49.14
	SSP245	SSP370	0.74	2.02E-07	34.21	36.21
	SSP245	SSP585	0.90	1.22E-17	45.67	47.67
	SSP370	SSP585	0.64	7.49E-06	38.97	40.97
2081-2100	present	SSP126	0.61	2.66E-04	29.30	31.30
	present	SSP245	0.69	2.48E-04	21.64	23.64
	present	SSP370	0.66	1.34E-05	34.25	36.25
	present	SSP585	0.45	7.08E-03	31.98	33.98
	present	COMBINED	0.61	4.36E-04	27.34	29.34
	SSP126	SSP245	0.78	1.01E-08	35.56	37.56
	SSP126	SSP370	0.86	2.15E-14	44.54	46.54
	SSP126	SSP585	0.77	2.12E-10	45.00	47.00
	SSP245	SSP370	0.71	1.47E-09	51.83	53.83
	SSP245	SSP585	0.56	1.97E-04	37.87	39.87
	SSP370	SSP585	0.81	2.36E-13	49.64	51.64

Supplementary Table 4. List of predictor variables hypothesized to affect plant biogeographic boundaries.

Code	Variable description	Resolution	References
Tectonic separation	Tectonic movements		88
ELEV	Topography	5 arcmin	58
VELOCITY	Past climate velocity during the late Quaternary	5 arcmin	40
BIO1	Annual Mean Temperature	5 arcmin	58
BIO2	Mean Diurnal Range (Mean of monthly (max temp - min temp))	5 arcmin	58
BIO3	Isothermality (BIO2/BIO7) (×100)	5 arcmin	58
BIO4	Temperature Seasonality (standard deviation ×100)	5 arcmin	58
BIO5	Max Temperature of Warmest Month	5 arcmin	58
BIO6	Min Temperature of Coldest Month	5 arcmin	58
BIO7	Temperature Annual Range (BIO5-BIO6)	5 arcmin	58
BIO8	Mean Temperature of Wettest Quarter	5 arcmin	58
BIO9	Mean Temperature of Driest Quarter	5 arcmin	58
BIO10	Mean Temperature of Warmest Quarter	5 arcmin	58
BIO11	Mean Temperature of Coldest Quarter	5 arcmin	58
BIO12	Annual Precipitation	5 arcmin	58
BIO13	Precipitation of Wettest Month	5 arcmin	58
BIO14	Precipitation of Driest Month	5 arcmin	58
BIO15	Precipitation Seasonality (Coefficient of Variation)	5 arcmin	58
BIO16	Precipitation of Wettest Quarter	5 arcmin	58
BIO17	Precipitation of Driest Quarter	5 arcmin	58
BIO18	Precipitation of Warmest Quarter	5 arcmin	58
BIO19	Precipitation of Coldest Quarter	5 arcmin	58

Supplementary Note 1

Climate change alters the future of natural floristic regions of deep evolutionary origins – ODMAP Protocol –

Samuel Minev-Benzecry , Barnabas Daru

2024-09-28

Overview

Authorship

Contact : bdaru@stanford.edu

Model objective

Model objective: Forecast and transfer

Target output: Projections of patterns of vascular plant diversity

Focal Taxon

Focal Taxon: Vascular plants

Location

Location: Global

Scale of Analysis

Spatial extent: -180, 180, -90, 90 (xmin, xmax, ymin, ymax)

Spatial resolution: 5-arc minute (~9 km)

Temporal extent: Present-day conditions (1970-2000) and four future climate scenarios (T1: 2021-2040, T2: 2041-2060, T3: 2061-2080, and T4: 2081-2100) based on MIROC6 and four Shared Socioeconomic Pathways (SSP 126, 245, 370 and 585). These pathways represent varying levels of climate mitigation, ranging from strong mitigation (SSP126) to moderate (SSP245 and SSP370) and high emissions (SSP585) scenarios

Boundary: natural

Biodiversity data

Observation type: range map, standardised monitoring data

Response data type: point occurrence, presence-only, richness

Predictors

Predictor types: climatic, habitat, topographic

Hypotheses

We hypothesize that if species' climatically suitable habitats contract under worsening climate conditions, β-diversity will likely increase, leading to the compositional differentiation of biogeographic regions. Conversely, in areas where species ranges expand to colonize new climatically suitable areas, β-diversity will decrease, and the composition of species assemblages will experience homogenization across geographic space. Climate change can also redefine biogeographic boundaries by rearranging species distributions without changes in their number. Altogether, we predict that climate change can homogenize, differentiate, and redefine biogeographic regions resulting in different biogeographic regions than we see today.

Assumptions

1) Biologically meaningful climatic predictors for plant species were used in the models.

2) Individual SDM were produced and stacked into unique layers depicting the global distribution and diversity patterns of vascular plant species.

3) The performance of the individual models was high across the 3 algorithms, both in cross-validation and the final predictions.

4) The integration of dispersal constraints using spherical Brownian motion model along with information on the natural habitat of species further improved performance

Algorithms

Modelling techniques: maxent

Model complexity: We used maximum entropy (MaxEnt v.3.4.3) to model plant species distributions. MaxEnt is not computationally expensive and has been shown to outperform other algorithms in modeling species distributions for computational efficiency especially when

dealing with a huge number of species spanning hundreds of thousands of species as in this study and is robust for modelling distributions for species with relatively few occurrence records. Model averaging: We generated five sets of models for each species and took the median to account for uncertainties across different model runs.

Workflow

We used maximum entropy (MaxEnt v.3.4.3) to model plant species distributions. MaxEnt is not computationally expensive and has been shown to outperform other algorithms in modeling species distributions for computational efficiency especially when dealing with a huge number of species spanning hundreds of thousands of species as in this study and is robust for modelling distributions for species with relatively few occurrence record. Predictor variables for the modeling were downloaded from WorldClim v.2.1 at a spatial grain resolution of 5-arcmin (equivalent to 10 km at the equator) for present-day conditions (1970-2000) and four future climate scenarios (T1: 2021-2040, T2: 2041-2060, T3: 2061-2080, and T4: 2081-2100) based on MIROC6 and four Shared Socioeconomic Pathways (SSP 126, 245, 370 and 585). These pathways represent varying levels of climate mitigation, ranging from strong mitigation (SSP126) to moderate (SSP245 and SSP370) and high emissions (SSP585) scenarios. We considered 20 predictor variables which are hypothesized to be important for plant distributions and diversity in previous studies. From these predictor variables, we removed areas corresponding to inland waters, i.e., lakes (using vector polygons from https://naturalearthdata.com). Variance Inflation Factor (VIF) was calculated among predictor pairs to remove highly autocorrelated predictors using the R package usdm version 2.1-6. Plant occurrence records used for the modeling were compiled from the Global Biodiversity Information Facility (GBIF, https://doi.org/10.15468/dl.jqqjba, accessed 2 June 2024) using the query term “Tracheophyta”. This yielded 454 million records from 14,405 published datasets. However, we previously showed that raw point occurrences of plants suffer from inherent coverage gaps and sampling biases that can hinder their ability to accurately represent global biodiversity patterns. To this end, we used a multi-step workflow to address these limitations as follows: (i) Source data: The raw occurrence data used to produce the species’ range polygons were obtained from GBIF (https://doi.org/10.15468/dl.jqqjba, accessed 2 June 2024). (ii) Data cleaning: These records were thoroughly cleaned by matching species names from the GBIF

occurrences to those in the World Checklist of Vascular Plants (WCVP) and keeping only verified names from WCVP. At the same time, the point records were refined to capture native distributions by intersecting them with WCVP's native range maps of vascular plants within country borders and retaining points that overlap WCVP's range maps. (iii) Polygon maps: After data cleaning, we converted the point records into polygon maps by modeling with alpha hulls using the R package rangeBuilder v.2.1. We cropped each species' polygon map to land areas using a basemap from naturalearth (https://naturalearthdata.com). Finally, we systematically sampled these polygon maps to generate 500 points per species for input into the species distribution model (SDM) as in previous studies rather than using the raw and biased point occurrences. (iv) Species-specific dispersal rate: We incorporated a partial-dispersal model to prevent erroneous predictions in suitable but unoccupied areas. Specifically, we calculated species-specific dispersal rates using a spherical Brownian motion model (SBM) implemented with the R package castor v.1.7.10. Unlike the widely used Brownian Motion models of continuous trait evolution that encode geographic locations in orthogonal space, the SBM model quantifies the dispersal of a clade over time as a diffusion-like process based on a single diffusion coefficient D, while accounting for Earth's spherical geometry. The SBM model was fitted using the function fit_sbm_const in the R package castor v.1.7.10. (v) Calibration area: The resulting dispersal rate, defined as the expected dispersal distance traversed by a species in a year (expressed in km/year), was used to define calibration areas (i.e., training areas) for modelling species distributions for each species across different timeframes. This was achieved by buffering the dispersal rates around the alpha hull polygons of each species and intersecting the buffered zones with maps of the terrestrial ecoregions of the world overlapped by the species to predict habitat suitability of each species. This latter step was intended as an additional fine-tuning process to allow us capture the natural habitats of each species based on their overlap with the ecoregions. (vi) Background points: We generated background points as a function of global plant sampling intensity to account for the biased sampling in the input occurrence records using spatial kernel density estimation and probabilistic sampling of 10,000 background points for each species within their calibration areas. (vii) Species distribution modeling: Species distribution modeling was conducted to estimate species distributions based on environmental conditions that correlate with known occurrences, and calibrated to species' realized niche based on the calibration area defined using the species-specific dispersal rates. From the occurrence

data as input, we used a 75% random sample for model development, while retaining the remaining 25% sample for model evaluation. For each species, we built models using a combination of hyperparameters in terms of the feature classes and regularization multiplier settings in MaxEnt v.3.4.3 as follows: linear, threshold, and hinge responses, and tested a set of regularization multiplier values (2, 5, 10, 15, 20) under a 5-folds cross-validation framework. Our models were predicted over each species' occurrences as a function of present-day bioclimatic variables and using these combinations of settings on a continuous scale between 0 and 1 using the sdm function in phyloregion v.1.0.9. We generated five sets of models for each species and took the median to account for uncertainties across different model runs. While ensemble methods that integrate and average predictions from multiple models are valuable, this can be computationally expensive and impractical for large datasets as in our study. With hundreds of thousands of species spanning five time horizons and four climate scenarios, creating ensembles for each would be computationally expensive and time-consuming. Moreover, if the individual models within the ensemble are highly similar, the ensemble may not provide much additional benefit compared to a single model. Therefore, we decided to run each model five times and take the median as a more efficient approach. For future climate scenarios, we modelled plant distributions as a function of present climate variables, and then used these models to predict plant distributions at new values of climate under different future scenarios for T1–T4 and SSP126, SSP245, SSP370, and SSP585. The model prediction consisted of a range map stored in raster format at a 5-arc minute grid cell resolution. The suitability of the models for each species was converted to binary presences by using the 95% quantile of the suitability values extracted from the underlying occurrences records as presence threshold. The final dataset contains range maps for 189,269 species, for the present, and four future time horizons each with four SSPs resulting in a total of 3,217,573 range maps for the analysis of biogeographical regionalization under climate change.

Software

All analyses were performed in R (R Development Core Team, 2024) using packages phyloregion v.1.0.9, rangeBuilder v.2.1, usdm version 2.1-6, MaxEnt v.3.4.3, castor v.1.7.10

Code availability: https://doi.org/10.5061/dryad.xd2547dqc

Data availability: Dataset is archived on Dryad at https://doi.org/10.5061/dryad.xd2547dqc.

Data

Biodiversity data

Taxon names: All vascular plants of the world

Taxonomic reference system: Plant occurrence records used for the modeling were compiled from the Global Biodiversity Information Facility (GBIF, https://doi.org/10.15468/dl.jqqjba, accessed 2 June 2024), using the query term “Tracheophyta”.

Ecological level: species

Data sources: Plant occurrence records used for the modeling were compiled from the Global Biodiversity Information Facility (GBIF, https://doi.org/10.15468/dl.jqqjba, accessed 2 June 2024) using the query term “Tracheophyta”.

Sample size: This yielded 402 million records from 11,517 published datasets.

Scaling: Polygon maps: After data cleaning, we converted the point records into polygon maps by modeling with alpha hulls using the R package rangeBuilder v.2.1. We cropped each species' polygon map to land areas using a basemap from naturalearth (https://naturalearthdata.com). Finally, we systematically sampled these polygon maps to generate 500 points per species for input into the species distribution model (SDM) as in previous studies rather than using the raw and biased point occurrences. Species-specific dispersal rate: We incorporated a partial-dispersal model to prevent erroneous predictions in suitable but unoccupied areas. Specifically, we calculated species-specific dispersal rates using a spherical Brownian motion model (SBM) implemented with the R package castor v.1.7.10. Unlike the widely used Brownian Motion models of continuous trait evolution that encode geographic locations in orthogonal space, the SBM model quantifies the dispersal of a clade over time as a diffusion-like process based on a single diffusion coefficient D, while accounting for Earth's spherical geometry. The SBM model was fitted using the function fit_sbm_const in the R package castor v.1.7.10.

Cleaning: These records were thoroughly cleaned by matching species names from the GBIF occurrences to those in the World Checklist of Vascular Plants (WCVP) and keeping only verified names from WCVP. At the same time, the point records were refined to capture native distributions by intersecting them with WCVP's native range maps of vascular plants within country borders and retaining points that overlap WCVP's range maps.

Background data: We generated background points as a function of global plant sampling intensity to account for the biased sampling in the input occurrence records using spatial kernel density estimation and probabilistic sampling of 10,000 background points for each species within their calibration areas.

Data partitioning

Training data: From the occurrence data as input, we used a 75% random sample for model development, while retaining the remaining 25% sample for model evaluation.

Validation data: For each species, we built models using a combination of hyperparameters in terms of the feature classes and regularization multiplier settings in MaxEnt v.3.4.3 as follows: linear, threshold, and hinge responses, and tested a set of regularization multiplier values (2, 5, 10, 15, 20) under a 5-folds cross-validation framework.

Predictor variables

Predictor variables: Predictor variables for the modeling were downloaded from WorldClim v.2.1 at a spatial grain resolution of 5-arcmin (equivalent to ~9 km at the equator) for present-day conditions (1970-2000) and four future climate scenarios (T1: 2021-2040, T2: 2041-2060, T3: 2061-2080, and T4: 2081-2100) based on MIROC6 and four Shared Socioeconomic Pathways (SSP 126, 245, 370 and 585). These pathways represent varying levels of climate mitigation, ranging from strong mitigation (SSP126) to moderate (SSP245 and SSP370) and high emissions (SSP585) scenarios. We considered 20 predictor variables which are hypothesized to be important for plant distributions and diversity in previous studies. From these predictor variables, we removed areas corresponding to inland waters, i.e., lakes (using vector polygons from https://naturalearthdata.com). Variance Inflation Factor (VIF) was calculated among predictor pairs to remove highly autocorrelated predictors using the R package usdm version 2.1-6.

Data sources: WorldClim v.2.1

Spatial extent: -180, 180, -90, 90 (xmin, xmax, ymin, ymax)

Spatial resolution: 5-arcmin (equivalent to ~9 km at the equator)

Coordinate reference system: WGS84

Temporal extent: Present-day conditions (1970-2000) and four future climate scenarios (T1: 2021-2040, T2: 2041-2060, T3: 2061-2080, and T4: 2081-2100) based on MIROC6 and four

Shared Socioeconomic Pathways (SSP 126, 245, 370 and 585). These pathways represent varying levels of climate mitigation, ranging from strong mitigation (SSP126) to moderate (SSP245 and SSP370) and high emissions (SSP585) scenarios

Transfer data

Spatial extent: -180, 180, -90, 90 (xmin, xmax, ymin, ymax)

Spatial resolution: 5-arcmin (equivalent to ~9 km at the equator)

Models and scenarios: Present-day conditions (1970-2000) and four future climate scenarios (T1: 2021-2040, T2: 2041-2060, T3: 2061-2080, and T4: 2081-2100) based on MIROC6 and four Shared Socioeconomic Pathways (SSP 126, 245, 370 and 585). These pathways represent varying levels of climate mitigation, ranging from strong mitigation (SSP126) to moderate (SSP245 and SSP370) and high emissions (SSP585) scenarios.

Model

Variable pre-selection

Variable pre-selection: Predictor variables for the modeling were downloaded from WorldClim v.2.1 at a spatial grain resolution of 5-arcmin (equivalent to ~9 km at the equator) for present-day conditions (1970-2000) and four future climate scenarios (T1: 2021-2040, T2: 2041-2060, T3: 2061-2080, and T4: 2081-2100) based on MIROC6 and four Shared Socioeconomic Pathways (SSP 126, 245, 370 and 585). We considered 20 predictor variables which are hypothesized to be important for plant distributions and diversity in previous studies. From these predictor variables, we removed areas corresponding to inland waters, i.e., lakes (using vector polygons from https://naturalearthdata.com).

Multicollinearity

Multicollinearity: Variance Inflation Factor (VIF) was calculated among predictor pairs to assess multicollinearity among predictors using the R package usdm version 2.1-6.

Model settings

Model settings (extrapolation): For each species, we built models using a combination of hyperparameters in terms of the feature classes and regularization multiplier settings in MaxEnt

v.3.4.3 as follows: linear, threshold, and hinge responses, and tested a set of regularization multiplier values (2, 5, 10, 15, 20) under a 5-folds cross-validation framework.

Model estimates

Coefficients: Our models were predicted over each species' occurrences as a function of present-day bioclimatic variables and using these combinations of settings on a continuous scale between 0 and 1 using the sdm function in phyloregion v.1.0.9.

Model selection - model averaging - ensembles

Model selection: We generated five sets of models for each species and took the median to account for uncertainties across different model runs.

Threshold selection

Threshold selection: The suitability of the models for each species was converted to binary presences by using the 95% quantile of the suitability values extracted from the underlying occurrences records as presence threshold.

Assessment

Performance statistics

Performance on training data: AUC, TSS, Boyce Index

Performance on validation data: AUC, TSS, Boyce Index

Performance on test data: AUC, TSS, Boyce Index

Prediction

Prediction output

Prediction unit: Species richness, community turnover and areas of refugia.

Data availability: https://doi.org/10.5061/dryad.xd2547dqc
