# Supplementary material for: Climate change alters the future of natural floristic regions of deep evolutionary origins
Source: Nat Commun. 2024 Nov 2;15:9474. doi: 10.1038/s41467-024-53860-8 (PMC11531475; doi:10.1038/s41467-024-53860-8)
Supplement: Supplementary file 2 — Reporting Summary [file 41467_2024_53860_MOESM2_ESM.pdf]

nature portfolio

Corresponding author(s): Barnabas Daru

Last updated by author(s): Sep 28, 2024

Reporting Summary

Nature Portfolio wishes to improve the reproducibility of the work that we publish. This form provides structure for consistency and transparency in reporting. For further information on Nature Portfolio policies, see our Editorial Policies and the Editorial Policy Checklist.

Statistics

For all statistical analyses, confirm that the following items are present in the figure legend, table legend, main text, or Methods section.

n/a	Confirmed	
☐	☒	The exact sample size (*n*) for each experimental group/condition, given as a discrete number and unit of measurement
☒	☐	A statement on whether measurements were taken from distinct samples or whether the same sample was measured repeatedly
☐	☒	The statistical test(s) used AND whether they are one- or two-sided<br>*Only common tests should be described solely by name; describe more complex techniques in the Methods section.*
☐	☒	A description of all covariates tested
☒	☐	A description of any assumptions or corrections, such as tests of normality and adjustment for multiple comparisons
☐	☒	A full description of the statistical parameters including central tendency (e.g. means) or other basic estimates (e.g. regression coefficient) AND variation (e.g. standard deviation) or associated estimates of uncertainty (e.g. confidence intervals)
☐	☒	For null hypothesis testing, the test statistic (e.g. *F*, *t*, *r*) with confidence intervals, effect sizes, degrees of freedom and *P* value noted<br>*Give P values as exact values whenever suitable.*
☒	☐	For Bayesian analysis, information on the choice of priors and Markov chain Monte Carlo settings
☒	☐	For hierarchical and complex designs, identification of the appropriate level for tests and full reporting of outcomes
☐	☒	Estimates of effect sizes (e.g. Cohen's *d*, Pearson's *r*), indicating how they were calculated

Our web collection on statistics for biologists contains articles on many of the points above.

Software and code

Policy information about availability of computer code

Data collection	Plant occurrence records used for the modeling were obtained directly from the Global Biodiversity Information Facility (GBIF, https://www.gbif.org/). No software was used to collect the data.
Data analysis	Data was analyzed using the following R packages: rangeBuilder v.2.1, castor v.1.7.10, MaxEnt v.3.4.3, phyloregion v.1.0.9, V.PhyloMaker2 v.0.1.0, terra v.1.7-55, sabre v.0.4.3, SpatialPack v.0.4, rgplates v.0.4.0, spdep v.1.3-1, hglm v.2.2-1, compute.es v.0.2-5

For manuscripts utilizing custom algorithms or software that are central to the research but not yet described in published literature, software must be made available to editors and reviewers. We strongly encourage code deposition in a community repository (e.g. GitHub). See the Nature Portfolio guidelines for submitting code & software for further information.

Data

Policy information about availability of data

All manuscripts must include a data availability statement. This statement should provide the following information, where applicable:

- Accession codes, unique identifiers, or web links for publicly available datasets
- A description of any restrictions on data availability
- For clinical datasets or third party data, please ensure that the statement adheres to our policy

Data availability. Plant occurrence records used for the modeling were downloaded from the Global Biodiversity Information Facility (GBIF, https://www.gbif.org/), accessed on 15 May 2023 using the query term “Tracheophyta”. From these records, we generated range maps using a combination of biodiversity informatics and

species distribution modeling, and the dataset is archived on Dryad at https://doi.org/10.5061/dryad.xd2547dqc. The phylogenetic tree used for the analysis is a published phylogeny that is already available in public repositories79. Specifically, the plant phylogeny was downloaded from Smith & Brown. Source data are provided with this paper.

Code availability. All scripts and code necessary to repeat the analyses described here have been made available in the new R package phyloregion.

Research involving human participants, their data, or biological material

Policy information about studies with human participants or human data. See also policy information about sex, gender (identity/presentation), and sexual orientation and race, ethnicity and racism.

Reporting on sex and gender	Not applicable in this study
Reporting on race, ethnicity, or other socially relevant groupings	Not applicable in this study
Population characteristics	Not applicable in this study
Recruitment	Not applicable in this study
Ethics oversight	Not applicable in this study

Note that full information on the approval of the study protocol must also be provided in the manuscript.

Field-specific reporting

Please select the one below that is the best fit for your research. If you are not sure, read the appropriate sections before making your selection.

☐ Life sciences ☐ Behavioural & social sciences ☒ Ecological, evolutionary & environmental sciences

For a reference copy of the document with all sections, see nature.com/documents/nr-reporting-summary-flat.pdf

Ecological, evolutionary & environmental sciences study design

All studies must disclose on these points even when the disclosure is negative.

Study description	We explored shifts in plant biogeographic regions under climate change using species distribution modeling under alternative climate change scenarios. Our models were constructed based on the standard protocol for reporting species distribution models using the ODMAP (Overview, Data, Model, Assessment and Prediction) protocol, along with open-source data and codes for scientific reproducibility. We used maximum entropy (MaxEnt v.3.4.3) to model plant species distributions. MaxEnt is not computationally expensive and has been shown to outperform other algorithms in modeling species distributions for computational efficiency especially when dealing with a huge number of species spanning hundreds of thousands of species as in this study and is robust for modelling distributions for species with relatively few occurrence records. Predictor variables for the modeling were downloaded from WorldClim v.2.1 at a spatial grain resolution of 5-arcmin (equivalent to ~9 km at the equator) for present-day conditions (1970-2000) and four future climate scenarios (T1: 2021-2040, T2: 2041-2060, T3: 2061-2080, and T4: 2081-2100) based on MIROC6 and four Shared Socioeconomic Pathways (SSP 126, 245, 370 and 585). These pathways represent varying levels of climate mitigation, ranging from strong mitigation (SSP126) to moderate (SSP245 and SSP370) and high emissions (SSP585) scenarios. We considered 20 predictor variables which are hypothesized to be important for plant distributions and diversity in previous studies. From these predictor variables, we removed areas corresponding to inland waters, i.e., lakes (using vector polygons from https://naturalearthdata.com). Variance Inflation Factor (VIF) was calculated among predictor pairs to remove highly autocorrelated predictors using the R package usdm version 2.1-6.
Research sample	Plant occurrence records used for the modeling were compiled from the Global Biodiversity Information Facility (GBIF, https://www.gbif.org/), accessed on 15 May 2023 using the query term “Tracheophyta”. This yielded 402 million records from 11,517 published datasets.
Sampling strategy	We used species distribution models to analyze 402 million occurrence records for vascular plants resulting in individual species-level native range maps for 189,269 species under present and future climatic projections throughout the twenty-first century. The selection of the 189,269 species reflects those with successfully modeled distributions that are consistent across different time horizons and climate scenarios.
Data collection	Plant occurrence records used for the modeling were compiled from the Global Biodiversity Information Facility (GBIF, https://www.gbif.org/), accessed on 15 May 2023 using the query term “Tracheophyta”. This yielded 402 million records from 11,517 published datasets.
Timing and spatial scale	The plant occurrence records were downloaded for contemporary time periods and at a global scale.
Data exclusions	No data was excluded. Instead, the occurrence records were thoroughly cleaned by matching species names from the GBIF

Data exclusions	occurrences to those in the World Checklist of Vascular Plants (WCVP) and keeping only verified names from WCVP. At the same time, the point records were refined to capture native distributions by intersecting them with WCVP's native range maps of vascular plants within country borders and retaining points that overlap WCVP's range maps.
Reproducibility	Data availability. Plant occurrence records used for the modeling were downloaded from the Global Biodiversity Information Facility (GBIF, https://www.gbif.org/), accessed on 15 May 2023 using the query term "Tracheophyta". From these records, we generated range maps using a combination of biodiversity informatics and species distribution modeling, and the dataset is archived on Dryad at https://doi.org/10.5061/dryad.xd2547dqc. The phylogenetic tree used for the analysis is a published phylogeny that is already available in public repositories. Specifically, the plant phylogeny was downloaded from Smith & Brown. Source data are provided with this paper.<br><br>Code availability. All scripts and code necessary to repeat the analyses described here have been made available in the new R package phyloregion.
Randomization	Not applicable in this study
Blinding	Not applicable in this study

Did the study involve field work? ☐ Yes ☒ No

Reporting for specific materials, systems and methods

We require information from authors about some types of materials, experimental systems and methods used in many studies. Here, indicate whether each material, system or method listed is relevant to your study. If you are not sure if a list item applies to your research, read the appropriate section before selecting a response.

Materials & experimental systems

n/a	Involved in the study
☒	☐ Antibodies
☒	☐ Eukaryotic cell lines
☒	☐ Palaeontology and archaeology
☒	☐ Animals and other organisms
☒	☐ Clinical data
☒	☐ Dual use research of concern
☐	☒ Plants

Methods

n/a	Involved in the study
☒	☐ ChIP-seq
☒	☐ Flow cytometry
☒	☐ MRI-based neuroimaging

Dual use research of concern

Policy information about dual use research of concern

Hazards

Could the accidental, deliberate or reckless misuse of agents or technologies generated in the work, or the application of information presented in the manuscript, pose a threat to:

No	Yes
☒	☐ Public health
☒	☐ National security
☒	☐ Crops and/or livestock
☒	☐ Ecosystems
☒	☐ Any other significant area

Experiments of concern

Does the work involve any of these experiments of concern:

No	Yes	
☒	☐	Demonstrate how to render a vaccine ineffective
☒	☐	Confer resistance to therapeutically useful antibiotics or antiviral agents
☒	☐	Enhance the virulence of a pathogen or render a nonpathogen virulent
☒	☐	Increase transmissibility of a pathogen
☒	☐	Alter the host range of a pathogen
☒	☐	Enable evasion of diagnostic/detection modalities
☒	☐	Enable the weaponization of a biological agent or toxin
☒	☐	Any other potentially harmful combination of experiments and agents

Plants

Seed stocks	Not applicable in this study
Novel plant genotypes	Not applicable in this study
Authentication	Not applicable in this study
